# Assessing the feasibility of a life history calendar to measure HIV risk and health in older South Africans

**DOI:** 10.1371/journal.pone.0226024

**Published:** 2020-01-15

**Authors:** Enid Schatz, Lucia Knight, Robert F. Belli, Sanyu A. Mojola

**Affiliations:** 1 Department of Public Health, University of Missouri, Columbia, Missouri, United States of America; 2 MRC/Wits Rural Health and Health Transitions Unit (Agincourt), School of Public Health, Faculty of Health Sciences, University of the Witwatersrand, Johannesburg, South Africa; 3 School of Public Health, University of the Western Cape, Belville, South Africa; 4 Department of Psychology, University of Nebraska, Lincoln, Nebraska, United States of America; 5 Department of Sociology, Princeton University, Princeton, New Jersey, United States of America; 6 Woodrow Wilson School of Public and International Affairs, Princeton University, Princeton, New Jersey, United States of America; 7 Office of Population Research, Princeton University, Princeton, New Jersey, United States of America; University of the Azores, PORTUGAL

## Abstract

Life history calendars capture patterns of behavior over time, uncovering transitions and trajectories. Despite the growing numbers of older persons living with HIV in southern Africa, little is known about how HIV testing and risk unfold in this population. Operationalizing a life course approach with the use of an innovative Testing and Risk History Calendar [TRHC], we collected pilot data on older South Africans’ risk and HIV testing. We found older persons were able to provide (1) reference points to facilitate recall over a 10-year period, (2) specifics about HIV tests during that decade, and (3) details that contextualize the testing data, such as living arrangements, relationships, and health status. Interviewer debriefing sessions after each interview captured information on context and links across domains. On a larger scale, the TRHC has potential to reveal pathways between sexual behavior, HIV testing and risk perception, and health at older ages.

## Introduction

Researchers have used life history calendars (LHCs) in a variety of contexts and populations to facilitate large-scale quantitative life course research, and more recently, as a qualitative technique that deepens learning about the life course [1–4]. The purpose of LHCs is to collect retrospective data in a number of domains through a fluid interview, using personal and social/political landmarks to facilitate recall [5,6]. Researchers have used LHCs to capture information on sexual relationships [7,8], health and employment among middle-aged and older adults [9–11], and important life transitions [8,12]. Research on sensitive topics suggests that LHCs can increase data quality, provide longitudinal data collected cross-sectionally, and reduce social desirability bias [7]. There is also evidence that LHCs produce high-quality data in diverse contexts, including in low- and middle-income countries [5,7,13]. In 2018, we piloted an LHC, the Testing and Risk History Calendar (TRHC), with 30 South Africans aged 50-plus. The TRHC included domains related to HIV testing, sexual relationships, and health care utilization. We used the TRHC to better understand the pathways between life domains that contribute to HIV risk as people age, as well as their interactions, and to assess the feasibility of collecting this information in a calendar format.

Currently, there are limited data on HIV testing and its correlation to health status and sexual relationships for adults transitioning to old age in HIV-endemic, African contexts. Although the HIV epidemic is aging, the focus of HIV data collection, interventions, and programming in Africa has centered on those aged 15–49 [14,15], with only a modest recent increased interest in populations outside this age range [16–18]. Overall, studies remain focused on younger adults (e.g., [19,20]).

South Africa has the largest number of individuals aging with HIV. In the early years of the epidemic, prevalence and mortality were highest among those aged 20–49 [21]. In the early 2000s, evidence began to emerge that there was a significant number of individuals aged 50-plus living with HIV, particularly men [22]. In the most recent data from South Africa, HIV prevalence is 17.2% among those age 50–64, and 20.6% among those aged 15–49 [23]. As we begin to see incidence rates drop at younger ages (from 1.36 in 2012 to 0.79 in 2017 among those 15–49) [23], prevalence rates are predicted to drop in these age groups, as well [23]. At the same time, antiretroviral treatment (ART) rollout allowed individuals to live longer with HIV, thereby shifting the epidemic and thus increasing prevalence at older ages; while incidence is lower in this age group, new cases are not insignificant [23,24]. This shift is likely to continue [23]: In 2017, the highest prevalence rate was among individuals in their 30s and 40s (as high as 39.4% among women 35–39, and 24.8% among men 45–49). As these individuals age on ART, the pool of infected possible sexual partners and risk of contracting HIV in one’s 50s and 60s will also grow, particularly with any non-adherence to ART. Although ART can reduce the risk of transmission [25], it can also increase infectiousness when individuals are non-adherent and their viral load increases.

HIV testing plays an important preventive role in two ways: (A) individuals who know their status can change their behavior, contributing to an HIV prevention cascade, and (B) individuals with positive results can begin treatment, a crucial step in the HIV care cascade [26]. Older persons present complications because not only are they less likely to have been tested for HIV, have talked to their partners about HIV [17], or be able to correctly identify transmission vectors [21], they also know less about ART [27]. The few studies on older persons’ risk behaviors suggest older persons often continue risky sexual practices [28–31] and, as part of aging, make relationship and residence changes that increase their exposure to risk [16,32,33]. Middle-aged and older adults often ignore risk reduction messages [34], even though they are at risk because of sexual violence [35,36], having sexual partners in high prevalence age groups [37,38], and having new partners after widowhood and divorce [37,39]. In recent survey data from rural South Africa, two thirds of respondents aged 40-plus reported multiple lifetime partners and among the 57% reporting sex in the last 2 years, 75% reported never using a condom [31].

The research on risk behavior, health, and HIV testing has used primarily cross-sectional survey data, which yields little information on how HIV testing and sexual behavior unfold over the latter half of the life course. Survey respondents are generally asked if they have ever been tested for HIV and the date of their last test. Because such cross-sectional surveys lack multiple time points, they cannot identify the order of events or build causal models between potential risk factors and HIV testing behavior [8,40]. There is even less precise information when HIV testing occurs and how it corresponds to changes in health status, or timing of screening and diagnosis for other conditions like non-communicable diseases (NCDs). Qualitative studies *have* examined testing norms and barriers and facilitators of testing. However, except for our own previous work [41,42], the studies primarily have been with younger populations [43,44].

When collecting HIV testing and risk data, a number of potential biases exist. First, recall bias can lead to telescoping the recency of the last test, heaping data within the last 12 months and making it appear that more individuals tested within the year than actually did [26]. Recall error can also be a challenge, particularly for older persons when faced with an autobiographical memory task regarding their life course observations and experiences [9,45,46]. Second, social desirability can bias reporting, in several ways. HIV negative adults have been found to over-report prior testing [26]. We have also found that, in a rural setting, older persons in focus group discussions are more likely to claim that people their age should be tested than older persons in this community report being tested in survey data from a similar time [42]. Reporting of sexual behaviour is also gendered: Men often over-report sexual partnerships and women under-report them [7,8,47]. Although less is known about whether the same is true for older men and women, gender roles and expectations in African settings suggest the direction of bias would be the same, even if the magnitude is not known [29,48]. Finally, current data on HIV testing includes very little on the social context surrounding testing decisions, sexual and marital histories, or broader health status [8]. The latter is an especially important omission because sub-Saharan Africa has an emerging NCD epidemic, particularly among older adults, which may mask HIV symptoms and reduce rather than increase the likelihood that providers suggest HIV testing [49,50].

The TRHC is a new, innovative way to overcome these data shortcomings and more deeply explore the links between risk, health, and testing over time among older Africans. The TRHC captures more complex information than traditional surveys by recording not only the occurrence of an event, but also timing, duration, and sequence across domains. In this way, the TRHC captures temporal order and enables causal inference [3,7,51], connecting HIV testing to key life events, such as the beginning or end of relationships, changes in health status, or the death of a family member. In addition, the TRHC provides longitudinal data more efficiently and at lower cost. Respondents situate events over time in relation to one another, which makes it more likely that the order of events is correct, even if actual dates are not exact [1,5,51]. Further, by ordering life events with contextualizing questions, the TRHC improves recall and reduces telescoping [3,7].

## Methods: Testing and Risk History Calendar (TRHC)

In 2017, we developed the TRHC, based on the Relationship History Calendar used with youth in Kenya [7], to understand when and why older South Africans do or do not test for HIV and how that relates to their histories of health and risk. Because older persons’ risk profiles might differ from younger persons, or be more protracted and distal, we looked at risk over a 10-year period. In addition, starting in 2011, there was a major increase in both HIV testing and ART availability in South Africa, so this is an salient decade to capture. The TRHC pilot data provide time-bound and time-varying data on HIV testing, sexual risk, and health at monthly intervals over a 10-year retrospective period for individuals aged 50-plus (i.e., those who are older or transitioning to older age).

**Fig 1:**
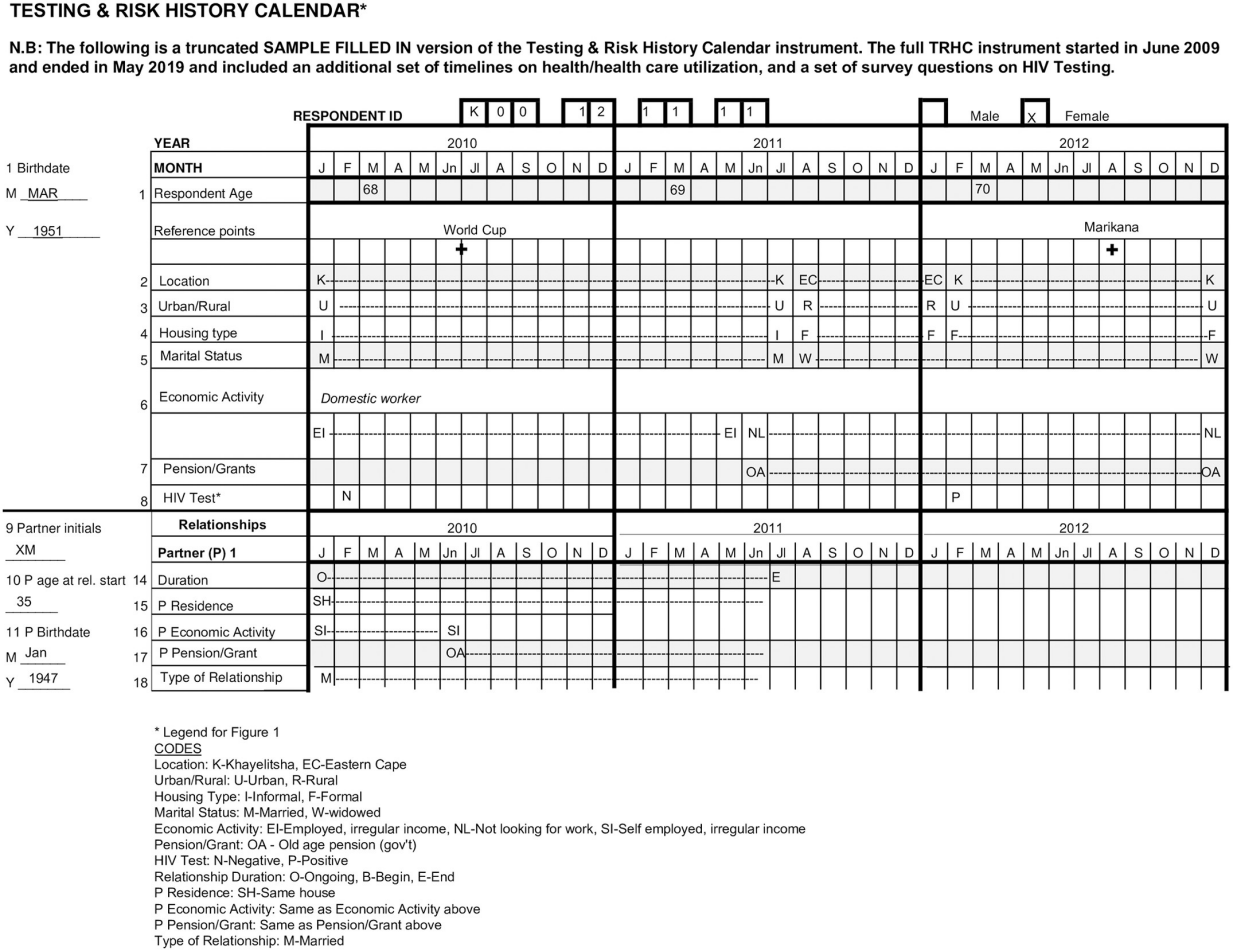
Visual example of filled-in Testing & Risk Calendar paper version fold-out grid.

The TRHC is formatted as a fold-out grid [1,3,7], with months across the top of the page and sociodemographic details and life domains down the left side of the page (Fig 1). Each month of an ongoing relationship is marked with a letter/line indicating relationship type and the key risks found to be correlated with older persons’ HIV acquisition and testing in prior studies [31,42,52]: frequency of sex, condom use consistency over the course of the relationship, and partner characteristics (i.e., age, residence, HIV status, etc.). Sociodemographic details including age, residence, education, marital status, employment, and social grant status were also collected, some in timeline form and some as discrete questions (See Fig 1). In the pilot, the three key life domains on the left side of the page included:

1. *Relationships and sexual risk*. Respondents recorded relationship dimensions and sexual behaviors for each relationship over the past ten years. This section allowed for the reporting of formal and informal partnerships, multiple concurrent partners, long- and short-term partnerships, casual sex, and all instances of sexual risk.
2. *Health status and health care utilization*: Respondents recorded non-communicable diseases, acute and infectious health events, and both routine and acute engagement with the health care system. This section allowed for tracking the timing of illness, diagnosis, and care, and the ways that engagement with the health system corresponds to HIV testing.
3. *HIV testing*: Respondents answered a set of survey questions about each HIV test that were in addition to placing each test in the last 10 years on the calendar. For each of the four most recent tests, the TRHC captures (a) where the test occurred, (b) motivation and reason for testing, (c) whether the respondent received test results, (d) what the tests results were, and (e) whether the respondent disclosed the result and/or (f) was linked to HIV care. If the respondent was linked to HIV care, the TRHC recorded the timing of ART initiation and adherence.

### Ethics

The study was approved by: Biomedical Science Research Ethics Committee of the University of the Western Cape (BM18/3/1 for Piloting of testing and risk history calendar), and Princeton University Institutional Review Board (IRB# 10798 for: HIV after 40 in Rural South Africa: Aging in the Context of an HIV/AIDS Epidemic), and the University of the Witwatersrand Human Research Ethic Committee (Medical) (Ref R14/49, M180290 for HIV after 40 in Rural South Africa: A follow up study). The University of Missouri Institutional Review Board (Health Sciences) signed a reliance agreement with UWC (#2011339-AA, Review #245851) and Princeton University (#2012518-AA, Review#241090) for data collection in Khayelitsha and Agincourt, respectively, and analysis of collected data. Consent was informed and written.

## Testing & Risk History Calendar pilot

In 2018, we piloted the TRHC with 18 individuals in an urban site and 12 in a rural site. The urban site is on the outskirts of the city of Cape Town, with a largely black African, isiXhosa-speaking population, several public health facilities, and a mix of formal and informal housing. The community has high levels of unemployment and most work is in the informal sector with low wages. Many of the older people in this community originally migrated from the Eastern Cape and still maintain links with and travel back and forth to that province.

The rural site is situated in Mpumalanga Province, South Africa. The community has low employment and high migration to urban areas for work, particularly among young men and women. In recent years, infrastructure projects have brought more paved roads and increased services such as a major grocery and department store chains to the area. The community has a wide wealth distribution, but the majority of people live in poverty and are dependent upon social grants and irregular remittances for household income [53].

### Recruitment

We purposively sampled older men and women (i.e., aged 50 years-plus) to reflect a range of ages and capture a significant proportion who had had an HIV test. *Urban site*. Because previous qualitative research suggested it would be challenging to find older people living with HIV, especially over aged 65 years and male [41], we used snowball sampling starting with individuals connected to social and health service organizations in which we had connections. We selected 18 respondents aged 48–73, including 5 men and 13 women. Of the respondents, 10 reported being HIV-negative, 6 reported living with HIV, and 2 never-tested/did not disclose. *Rural site*. At this site, another study on HIV among adults aged 40–60-plus was taking place and recruiting focus group participants from market places, community centers, and other community meeting locations. We recruited participants for our study who did not fit the age range for the other study’s focus groups. The interviewer also recruited participants from local villages by approaching older-looking persons. Altogether, we selected 12 respondents aged 50–81 years, 6 men and 6 women. Only one respondent reported living with HIV.

### Interview process

Trained research assistants conducted all of the interviews, which were designed to assess the feasibility of the calendar method for collecting data from older people. Compared to a standard questionnaire, the THRC interviews were less structured and guided by the identification of key reference points. To facilitate recall, we printed three public reference points on the TRHC: the World Cup in June and July 2010, the Marikana Massacre in August 2012, and Nelson Mandela’s death in December 2013. At the beginning of the interview, respondents added salient personal reference points to the calendar (e.g., birth of grandchildren, the death of family members, retirement, moving). Throughout, interviewers moved back and forth between domains to correct the timing of events, including HIV tests. For example, an interviewer might say, “You said your husband died in March of 2015, and you also said you had an HIV test in April of 2015. Was your HIV test in the month after your husband died?” The interviewer would then correct the date on the calendar according to the answer. In many cases, a discussion about one event, such as an illness, would trigger a memory about another event around the same time, for example a risky behavior. Throughout, respondents could add events to the calendar any time they were recalled. Interviewers also wrote margin notes with information that did not easily fit in the calendar format.

Each interview lasted approximately two hours. Each respondent took time to provide information about each partner and health condition from the past ten years. Women reported zero to one relationship (mode was zero) and zero to three health conditions (mode was two). Men reported one to three relationships (mode was two) and zero to three health conditions (mode was one).

### Debriefing process

We debriefed the interviewer after each interview, which allowed us to learn about any information pertinent to relationships, health and HIV testing that respondents shared but did not fit easily into the calendar format. During the debriefing, the interviewer specified how they recruited this respondent, explained any margin notes, and shared any ideas about how to improve the instrument. The person debriefing the interviewer took notes and attached those to each calendar.

## Results

### Reducing telescoping errors and recall bias

We expected the TRHC to reduce the likelihood of telescoping during recall [3] because it would allow respondents to anchor HIV, health, and sexual behaviors against social and personal landmarks. Indeed, in both the urban and rural contexts, older persons were able to recall and place on the TRHC when they had tested for HIV, the timing of sexual relationships, and information related to their health conditions, as well as the components of each relationship (e.g. condom use and the frequency of sex) that changed or were static over time. Nearly all respondents reported at least one HIV test in the last 10 years (27 out of 30). Of those who reported having an HIV test, 25% (7 of 27) reported a test in the last year (2018), 48% (13 of 27) reported their last test in the previous 1–2 years (2016–2017) (48%), and 26% (7 of 27) reported their last test was 3 or more years ago.

The debriefing notes further illustrate the anchoring value of the TRHC approach. A 53-year-old woman made a clear connection between one of her reference points and her HIV test. The debriefing notes read:

Points of reference: 2009—accused by community members of having HIV. Women in her community harassed her by calling her to their house where a group of women would accuse her of being HIV positive. They said she was denying her HIV status. She went to a clinic and spoke to a counselor to discuss what was happening. She tested for HIV and her results were negative. The counselor at the clinic told her to bring the ladies that were harassing her to the clinic. She brought two of the women to the clinic and the counselor told her them to stop the harassment and she does not have HIV. But this did not stop the harassment (Khayelitsha-16, age 53).

While this particular event was extreme, it is interesting to note the reasoning this respondent gave for each test, which the interviewer took note of:

Tested for HIV 5 times, once before 2008 and 3 times since. First test was in 2003 when she fell pregnant. Tested in 2003—tested when pregnant. Tested in 2007—because her sister tested positive (and is still alive on treatment). Tested in 2009 (this was due to the harassment) and disclosed to the entire community. Tested 2012—because she wanted to be tested. It was on her own initiative and disclosed to her partner. Tested 2017—at a routine health check-up and disclosed to her partner. All tests were negative and she always tests alone.

As these notes illustrate, the calendar provided information that would have been missed by the standard survey question “have you ever tested for HIV.” Using the calendar provided an opportunity, through debriefing, to gain insight into the motivations underlying the THRC interrelationships among events that involved respondents’ HIV testing histories. Such depth of understanding would be invisible in standard surveys that are not optimized to collect the temporal and thematic interrelationships among events, or qualitative contextual data.

### Reducing social desirability bias—Gender differences

On average, men reported having one to two partners over the 10-year period, which rarely overlapped. Only one man reported three partners. In other data from this context, 10–15% of men in this age bracket reported having two partners in the last two years [16,31]. In contrast, men in this study reported fewer partners (only two of our respondents reported two partners in the last two years). In surveys, when men do not have to account for actual relationships, they might be more likely to over-report the number of relationships due to social desirability [7].

For the most part, women reported zero partners in the past 10 years, other than husbands. Nine of the 19 women reported partnerships with a husband. The interviewers probed extensively to learn about extra-marital or post-marital relationships for those divorced or widowed (e.g., interviewers asked if respondents had a man who they used as “roll-on” or that they were “keeping for the future”), but women reported very few non-marital partners. It is unclear if this is because women are reluctant to report non-normative sexual behaviour, or are truly not having sex and finding new partners. Survey results from another study are similar: 36% of women in their 50s and 70% of women in their 60s reported no sexual partners in previous 2 years, and the remaining said they had had one relationship in the past 2 years, presumably their husbands [16].

One HIV-negative woman explains having no sexual partners in the last 10 years; the margin notes read:

The husband is no longer coming home. The last time he came home was in 2004. Since then she said she hasn’t had any sexual partner or other partner. She said that she’s still waiting him to come home. She still hears from him sometimes but he hasn’t actually come home in years… No HCU [health care utilization]. Last time she got sick was in 2006. She doesn’t go to the clinic at all, only went when she was at work and very ill, which was the time that they tested her for HIV (Agincourt-9, age 59).

A man who reported three current partners also expounded on the TRHC relationship questions:

Relationship—has currently 3 partners, but the first partner started in 2006, the other two are more recent in last year or so. He was just attracted to them. Never used condom with any of the 3. Doesn’t know if any of the current 3 partners have ever tested (Agincourt-12, age 54).

In sum, while the number of reported partnerships continue in the direction of known gendered social desirability bias [7], the need for detail on the TRHC appears to be tempering the men’s reports of relationships, but not increasing the women’s reports.

### Increasing causal inference

**Fig 2:**
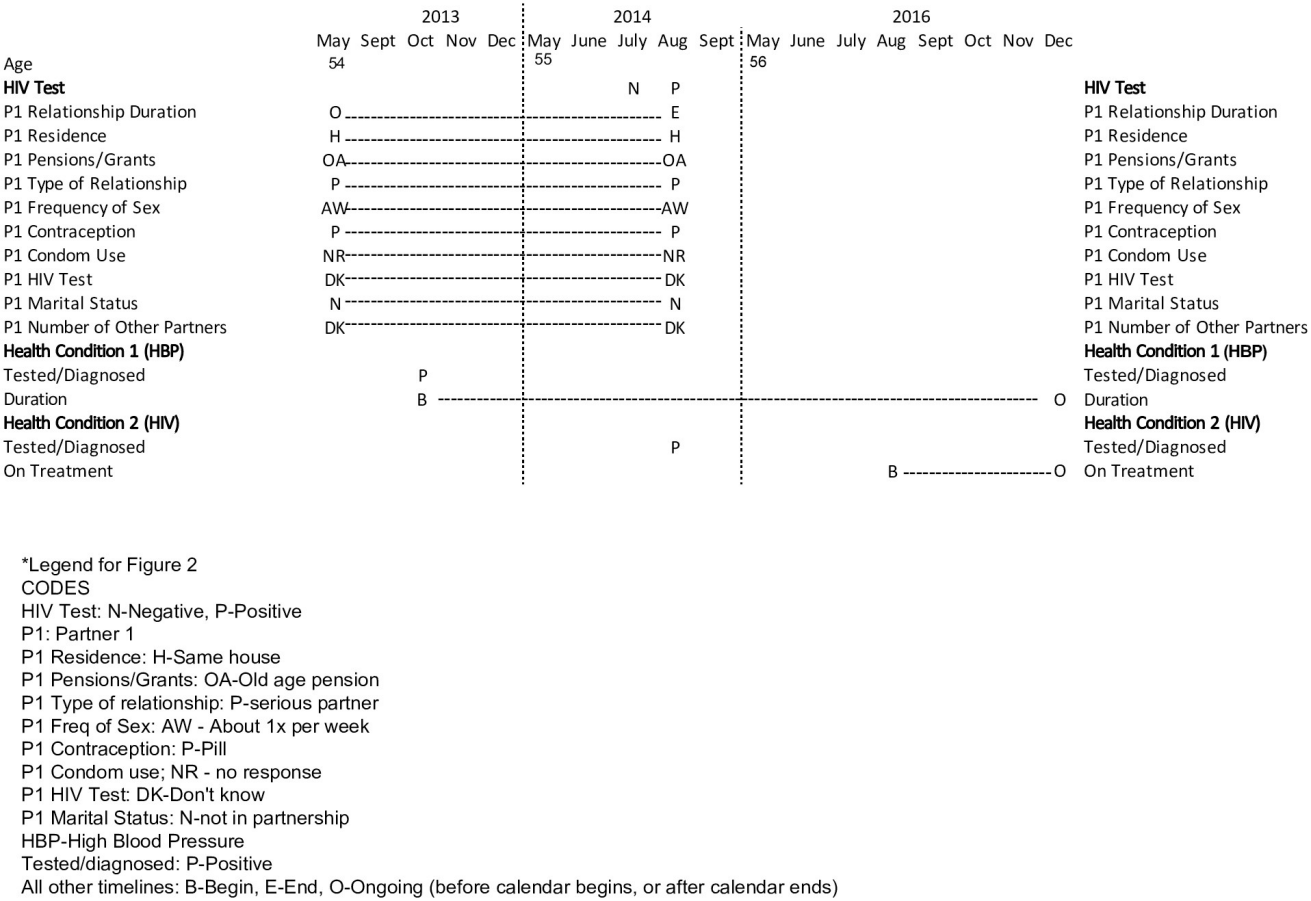
Abbreviated TRHC for Khayelitsha ID#06—Showing age, HIV testing, relationship and health condition related timelines (2013–2016, 2015 not shown).

The pilot findings from the TRHC enable some causal inferences to be drawn about the relationship between risk, health and HIV testing. By having a clearer sequence of events, and in some cases, notes specifying that one event directly impacted or led to another, there is less guessing about the order of events or how they may be related, or when opportunities for testing might have been missed. For example, the calendar events for HIV testing and NCD diagnosis are highlighted in Fig 2 for a 57-year-old female respondent that tested HIV positive in 2014. She reported a relationship that was ongoing at the start of the calendar, but ended by September 2014. She reported HIV tests in July and August 2014 and high blood pressure in October 2013.

The debriefing notes clarify why she was tested two months in a row.

Tested for HIV July 2014 and results were negative and tested one month later in August and tested positive. She did not know why they asked her to return in one month to be tested. She said she was not shocked and accepted it as any other illness. Her motivation to get tested was that her sister’s husband died of HIV. He did not disclose. It was only after his death that her sister found tablets. Her sister then tested and was positive. [The respondent was in a] relationship that began prior to 2008 and ended in 2014. They were cohabiting… she was diagnosed with HIV, soon after he left. They lived together in Khayelitsha and he had permanent income. They were a serious couple but there were times he did not come back from work, and would say transport broke or had to work overtime. At the time she believed him but now thinks that this is when he was having other sexual relations. Frequency of sex was 1/week. She was on the pill and not using condoms. She doesn’t know if he was ever tested for HIV.” (Khayelitsha-06, age 57).

Taken together, the calendar data and debriefing notes provide information both about risk behavior and the sequence of events. Despite having been connected to the health system for monthly high blood pressure treatment visits since October 2013, she did not report testing for HIV as part of these visits until she requested a test due to her sister’s husband’s death in July 2014 (not shown in Fig 2, there were no HIV tests listed prior to July 2014). Despite having risky sexual behaviour (sex weekly with a partner without condoms and whose HIV status she did not know), she did not say that this contributed to her inclination to test. Further, due to being tested prior to Universal Test and Treat, she only started ART in August 2016, nearly two years after her initial diagnosis.

**Fig 3:**
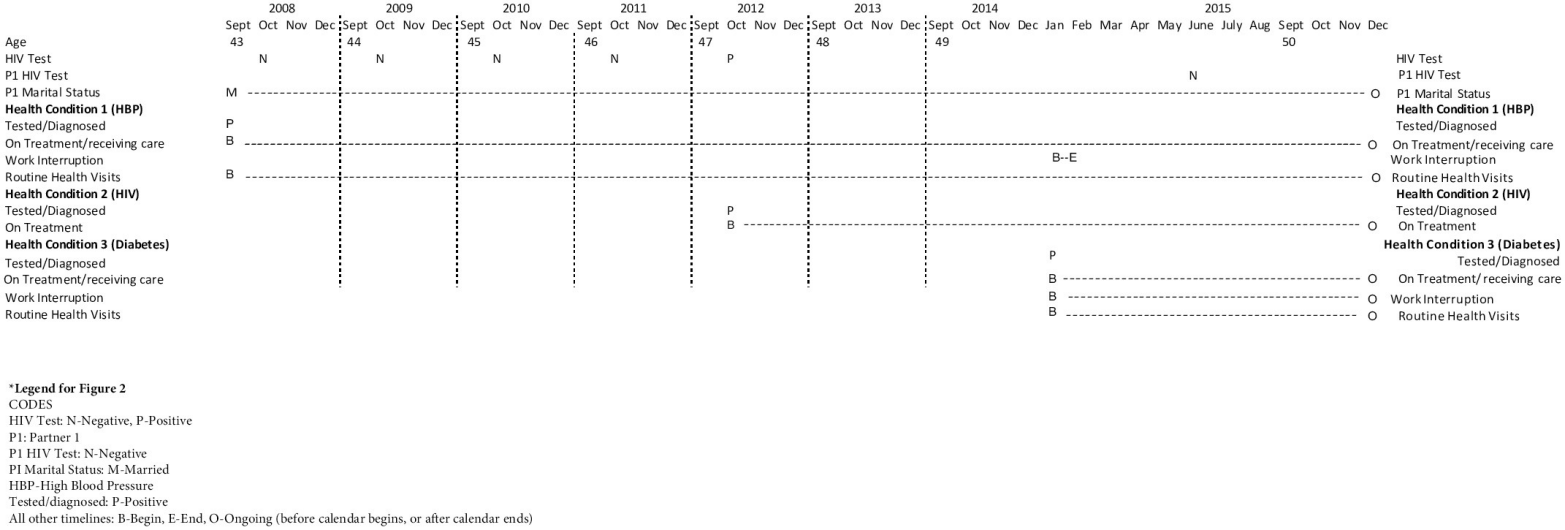
Abbreviated TRHC for Agincourt ID#02—Showing age, HIV testing, and select relationship status and health condition related timelines (2008–2015).

On the other hand, the TRHC data for a 53-year old woman from the rural site (Fig 3) shows a history of yearly testing (likely part of antenatal/reproductive health care) prior to turning 50. She was diagnosed with HIV in 2012 resulting in a connection to care and being initiated on ART at the time of the positive test. Her partner, to whom she was married, only tested (negative) for the first time in 2015. Further, while she also was in care for hypertension (diagnosed prior to 2008), in January 2015, while engaged in regular visits for HIV and hypertension treatment, she was diagnosed with diabetes, which caused more extensive work interruption than either her HIV or HBP diagnoses.

Although individual cases are not sufficient to assess trends, these type of data on a larger scale will have important implications for understanding the causal relationships between and timing of risk behaviors and perception, NCD diagnosis and engagement in care, and HIV testing.

### Providing context

A final advantage of the TRHC approach is that we expected it would add more information on the social context surrounding testing decisions that is lacking in existing survey data, such as the dynamism of sexual relationships over time [7,32], the potential importance of sexual and marital histories [8], and personal health and wellness [18]. The TRHC demonstrated the distinct advantage of being less structured and more informal than a standard survey interview, but not as free form as a qualitative in-depth interview. This format allowed interviewers to capture more information systematically on the calendar, as well as other information through margin notes that would have been lost altogether in a standard survey. In this way, the TRHC capitalized on the strengths of a qualitative approach while collecting quantitative data [2].

As exemplified above, margin comments and debriefing sessions provided contextual information. For example, in reference to why a 70-year-old woman had never tested for HIV, a margin note reads, ‘Don’t use condoms, because trust husband; doesn’t see any need to go and do a test; to her knowledge husband also hasn’t been tested. Trust him.’ (Khayelitsha-02, age 70). In contrast, margin notes for a 50-year-old man in the rural site indicated that he went for an HIV test because he did not trust his partner, ‘Had HIV test in 2013 after he divorced his wife. He found his wife cheating so he wanted to know his status and make sure he was still OK’ (Agincourt-10, age 50). Other brief margin notes provide context, such as the notes for a 59-year-old man, ‘thought he was very careful with behavior so was shocked when found out he was positive in 2015’ and ‘Not a man who sleeps around’ (Khayalitsha-04, age 59).

The margin comments and information from debriefing sessions provided context and/or reasoning for decision-making around key events captured in the TRHC—timing of relationships, frequency of and protection during sex, and connections between relationships and HIV testing. These are important data points in and of themselves, but they also provide the context necessary to build causal models and interpret statistical models. Because these data are embedded in individual, chronological stories, researchers can make decisions about the meaning of particular variables and patterns, allowing for a rich interpretation of the timing and relationship between events for any individual and more broadly if similar patterns are found in the aggregate.

## Discussion

The TRHC provides an unusual breadth, with 10 years of retrospective, time-bound data and depth, with margin comments and debriefing notes that give context. With this, the THRC improves on some of the shortcomings of survey data [26], specifically by (1) reducing telescoping and recall error related to the timing of HIV tests; (2) improving the quality of data on the number and concurrency of relationships by asking relationship questions in a way that reduces social desirability bias, and (3) increasing our ability to build causal models around sexual behaviour, co-morbidities and HIV testing outcomes by having time-bound data and qualitative information about motivational drivers. The TRHC helps to measure and assess how HIV risk and vulnerability unfold over the life course, providing important longitudinal data generally not available, particularly for this population [16].

With respect to telescoping and recall error, the THRC demonstrates advantages that align with what methodological research would predict. Although the timing of events that respondents provide in the TRHC may not be exactly as it occurred in real life, there is strong evidence from other settings and projects that the ordering is likely to be correct even if the exact timing is at best, an approximation [5,13,46]. And, it does appear that the TRHC encourages respondents to anchor past HIV testing on a number of socio-political and personal reference points, which methodological research using LHC has shown increases the accuracy of retrospective reports [54–56].

Addressing social desirability bias is important because in survey data the bias often results in men over-reporting sexual partners and women under-reporting them. Although a study with a larger sample is needed to ensure the veracity of the information in the TRHC pilot, the net data on relationships appears as good, if not better, than recent survey data. In particular, the TRHC format allows for cross-checking information across domains and resolving any inconsistencies that are found [51], which is one means of reducing responses that are due to social desirability rather than actual events. Also, the fluid form of the TRHC interview allows interviewers to embed sensitive questions in a broader history of respondents’ lives [1,7], such as asking ‘were you in a relationship when you tested for HIV that time?’ or asking about HIV testing in relation to a partner passing away or suspecting a partner of having another sexual partner. These features may reduce social desirability bias [3], allowing us to get better information about older persons’ partnership and potentially risky sexual behavior [7]. Nonetheless, the ability of the TRHC to reduce social desirability bias may be gendered, since it appears that women are no more likely to report partnerships, particularly those outside of marriage, than in surveys in similar contexts [57].

Although this small pilot sample restricts the ability to assess trends and relationships at an aggregate level, the pilot demonstrates how the ordering on the calendar can help suggest causal relationships (or missed opportunities for testing), particularly with the addition of debriefing notes. The quantified TRHC data can be used to assess relationships between key variables that predict HIV testing behaviour, such as the beginning or end of partnerships or changes in health status. The TRHC allows us, for the first time, to assess whether individuals with higher risk behavior (e.g, starting new relationships, high frequency of sex without condoms) are also the individuals who are getting tested, and whether there are gender differences in partnership status prior to testing (e.g., death of a partner) or after testing (e.g,. partnerships ended after a positive test). It also allows us to see if individuals who are engaged in the health system for an NCD are any more likely to be routinely tested for HIV.

Finally, the pilot TRHC study makes clear several key revisions that will strengthen the instrument. First, while visually important, the paper fold-out grid was cumbersome for participants and interviewers to work with. We are in the very early stages of building an application for tablet computers in collaboration with Dr. Abu Mosa (University of Missouri, Department of Health Informatics) that will allow interviewers to collect data in analytic-friendly electronic files and allow respondents to see a calendar on which to post key dates, thus preserving the visual nature of the interview in a less cumbersome format. The application format will allow interviewers to include survey-type questions or prompts for particular events that we learned were too fine-grained for recall in a calendar format (e.g., medications taken, transportation to appointments, dates when partners were tested for HIV). Third, although margin comments and debriefing notes were key to understanding the contextual factors around HIV testing, relationships, and health, we know debriefing interviews are not possible at a larger scale. To address this, the application will include space for general “margin comments” and a series of questions similar to those asked by the project leaders during debriefing. Further, we will develop a tool to assess the quality of the debriefing notes that will contribute both to enriching the research and future quality improvement of the instrument. As these notes will now be inputted text, they will also be searchable and we will be able to extract and code these data. Together, the margin comments and debriefing notes will supply the additional context researchers need to select variables and build causal models. Finally, note that the application format can be designed with a metadata-based approach that customizes components of the calendar based on individual project requirements. The custom nature of the application will make it adaptable for research teams and social service organizations that have different needs for using a LHC.

Looking ahead, we will build on the pilot TRHC by using the refined instrument in a computer application format with a randomly sampled population to asses gendered trends in HIV testing over time, as well as the previously unstudied relationships between risk and testing in older Africans. Adding components to further identify key contextual and life course variables will also be important. For example, ethnicity is a salient marker of context in South Africa that captures both social relations (class composition, residential location, and age and sex structures) and cultural relations (language, religion, and social/marriage patterns); in future iterations, particularly those that include participants from a variety of ethnic groups, including ethnicity would enhance the life course approach. Capturing, synthesizing, and understanding the context and correlates of testing HIV behavior is particularly important for older persons because as they age out of high-risk age-groups, they also age out of routine testing. And, in comparison to younger persons, older persons have a longer lifetime of accumulated risk and history of testing to capture and understand. Thus, as the HIV epidemic becomes an epidemic of aging, standard survey instruments will not give us the answers we need. The TRHC is an innovative means of collecting higher-quality, retrospective, longitudinal data. Researchers and policy makers will need to decide where funds and resources should go to most effectively reduce risk and encourage earlier testing among persons aged 50-plus who are at risk for HIV; we argue that the TRHC would provide much needed data to answer core questions about HIV testing, health, and risk in an aging population.
